# Biting by *Anopheles funestus* in broad daylight after use of long-lasting insecticidal nets: a new challenge to malaria elimination

**DOI:** 10.1186/1475-2875-13-125

**Published:** 2014-03-28

**Authors:** Seynabou Sougoufara, Seynabou Mocote Diédhiou, Souleymane Doucouré, Nafissatou Diagne, Pape Mbacké Sembène, Myriam Harry, Jean-François Trape, Cheikh Sokhna, Mamadou Ousmane Ndiath

**Affiliations:** 1 Unité de Recherche sur les Maladies Infectieuses et Tropicales Emergentes, IRD198, UM63, CNRS7278, INSERMU1095, Aix-Marseille Université, Campus UCAD-IRD, BP 1386, CP 18524 Dakar, Sénégal; 2 Département de Biologie Animale, FST/UCAD, BP 5005 Dakar Fann, Sénégal; 3 Laboratoire Evolution, Génomes et Spéciation, Université Paris-Sud 11, 91198 Gif-sur-Yvette, Cedex, France; 4 Laboratoire de Paludologie, Campus International UCAD-IRD Hann, BP 1386 CP 18524 Dakar, Sénégal; 5 G4 International Group, Institut Pasteur International Network, Entomology Unit, Institute Pasteur of Bangui, BP 926 Bangui, Central African Republic

**Keywords:** Malaria, Anopheles, Resistance, Behaviour, Adaptation

## Abstract

**Background:**

Malaria control is mainly based on indoor residual spraying and insecticide-treated bed nets. The efficacy of these tools depends on the behaviour of mosquitoes, which varies by species. With resistance to insecticides, mosquitoes adapt their behaviour to ensure their survival and reproduction. The aim of this study was to assess the biting behaviour of *Anopheles funestus* after the implementation of long-lasting insecticidal nets (LLINs).

**Methods:**

A study was conducted in Dielmo, a rural Senegalese village, after a second massive deployment of LLINs in July 2011. Adult mosquitoes were collected by human landing catch and by pyrethrum spray catch monthly between July 2011 and April 2013. Anophelines were identified by stereomicroscope and sub-species by PCR. The presence of circumsporozoite protein of *Plasmodium falciparum* and the blood meal origin were detected by ELISA.

**Results:**

*Anopheles funestus* showed a behavioural change in biting activity after introduction of LLINs, remaining anthropophilic and endophilic, while adopting diurnal feeding, essentially on humans. Six times more *An. funestus* were captured in broad daylight than at night. Only one infected mosquito was found during day capture. The mean of day CSP rate was 1.28% while no positive *An. funestus* was found in night captures.

**Conclusion:**

Mosquito behaviour is an essential component for assessing vectorial capacity to transmit malaria. The emergence of new behavioural patterns of mosquitoes may significantly increase the risk for malaria transmission and represents a new challenge for malaria control. Additional vector control strategies are, therefore, necessary.

## Background

During the past decade, the control of malaria has made slow but steady progress, the overall mortality rate dropping by more than 25% since 2000 [1]. Behind the statistics and graphs, however, lies a great, needless tragedy, with the life of an African child taken every minute [2]. The strategic approaches to control malaria rely on antivectorial programmes, which include mainly indoor residual spraying and insecticide-treated bed nets. The effectiveness of these strategies depends on the susceptibility of the vector species to insecticides and their behaviour, ecology and population genetics.

In Africa, these front-line control tools are very efficient against the main vectors of malaria, including *Anopheles gambiae* and *Anopheles funestus*, which prefer to bite and rest indoors at night when people are in bed [3,4]. Both species have high capacity to transmit malaria parasites, because of their anthropophagic and endophilic characteristics, their longevity and their abundance [3]. Therefore, indoor interventions may protect people against infectious bites. These include reducing vector populations by mass killing, leading to a significant reduction in the lifespan, human contact and malaria sporozoite rate of *Anopheles* mosquitoes [5], and use of excito-repellent insecticides that cause mosquitoes to leave rooms for outdoors [6].

Many studies show that the effectiveness of these tools has been compromised by the emergence of *kdr* insecticide resistance, physiological and metabolic resistance of the vectors to insecticides [7-9]. Pyrethroid-treated bed nets are widely deployed in Africa, the estimated percentage of households with one impregnated net having increased from 3% in 2000 to 53% to 2012 [2]. The dramatic increase in *Anopheles* resistance may, however, reverse the effects of vector control programmes, leading to a resurgence of malaria morbidity in several parts of Africa [10,11].

The emergence of insecticide-resistant *Anopheles* mosquitoes is also associated with behavioural changes that lead the vector to avoid intra-domiciliary vector control tools. Formerly, the maximum of anophelines aggressiveness was typically observed in the middle of night but since the introduction of LLINs significant changes were observed. In 1990, Fontenille *et al.*[12] showed that the *An. funestus* biting peak occurred from 01:00 to 03:00 indoors and from 02:00 to 05:00 outdoors. Furthermore, in Tanzania, Russell and al [13] in 2011 reported a nocturnal activity of *An. funestus* before insecticide-treated use became widespread. No studies reported behavioural activities of *An. funestus* prior to implementation of strategies control. Indoors interventions, such as LLINs and IRS, have become an ideal tool to protect people from biting mosquitoes and the correct use of these tools decrease the burden of malaria transmission [14,15]. However, to ensure their survival mosquitoes adopt behavioural change and the best time to find host is the early hours of the night or at dawn. This form of resistance is an adaptive strategy, which allows the mosquitoes to avoid or circumvent control strategies [16-18].

Charlwood *et al.*[19] in Papua New Guinea reported that although many anophelines were killed after the introduction of impregnated nets, a portion was diverted outside. Females that have been diverted have a longer oviposition cycle and therefore tend to bite earlier the following evening. In Burkina Faso, Riehle *et al*. [20] identified a new subgroup, *An. gambiae* Goundry, which presumably bites only outside and is more susceptible to *Plasmodium* infection. The authors reported that this new subgroup would probably be selectively favored by indoor vector control measures.

The emergence of the new phenotypes of mosquitoes was also reported elsewhere, probably natural selected by changing environmental conditions that create new selection pressures [21] Moiroux *et al*. [22] reported that, in Benin, three years after universal coverage with LLINs, *An. funestus* showed substantial diurnal and early biting activity and more frequent outdoor biting. The behavioural effect on *An. funestus* may, therefore, be genetic or phenotypic adaptation to widespread use of LLINs and IRS. This change in mosquito biting habits could jeopardize the success of control operations. In addition to insecticide resistance, behavioural adaptation of mosquitoes that feed exclusively outdoors at early hours of the night or morning may affect the epidemiology of malaria transmission. The aim of this study was to investigate the population dynamics of mosquitoes in relation to their biting activity after prolonged use of insecticide-treated bed nets.

## Methods

### Study area

Studies of the relations between the host and the malaria vector have been conducted since 1990 in Dielmo [23-26], a village of 400 inhabitants located 280 km southeast of Dakar near the Nema River, which results in *Anopheles* larval proliferation all year round. Rainfalls occurs during a four-month period, from June to October. Two events made this village note worthy in the vector control programme: the first distribution of LLINs in July 2008, followed by a total renewal in July 2011 after a spectacular rebound of malaria [10].

### Mosquito collection

Adult mosquitoes were collected monthly between July 2011 and April 2013 by human landing catches (HLC) and pyrethrum spray catches (PSC). Hourly HLC were made on adult volunteers between 19:00 and 07:00 at two point of capture on three consecutive nights, with two collectors, one indoors and one outdoors, positioned at each site; this is referred to below as “standard catching at night” (SCN). On one of two point of capture, collected mosquitoes (one indoors and one outdoors) were continued until 11:00 during the third day in January-April 2013. Which are referred to as “new catching by day” (NCD) mosquitoes collected between 07:00 and 11:00. PSC were conducted in two rooms randomly selected among those in which no form of insecticide or repellent had been used during the previous week and which were different from those used for HLC. Deltamethrin (Yotox®) was sprayed inside the closed rooms for 30–45 seconds, and dead or immobilized mosquitoes were collected after 10 minutes. The points of collection were distributed randomly in the village.

### Field and laboratory processing

Anophelines were identified by the morphological identification keys of Gillies and De Meillon [3]. The human biting rate was estimated from the number of bites per person per night sampled by HLC. Endophagous rates were calculated as the proportion of the number of mosquitoes captured indoors among the total number of mosquitoes captured by HLC. The ovaries of female anophelines were dissected to determine the status parous/nulliparous [27]. The blood meal of blood-fed females captured by PSC were squashed on to Whatman No. 1 filter paper and tested by enzyme-linked immunosorbent assay (ELISA) to identify bovine, ovine, caprine (sheep and goat), equine (horse and donkey) or chicken origin as previously described [28]. The anthropophilic rate was calculated as the proportion of mosquitoes that fed exclusively on human blood among all fed mosquitoes. The detection of circumsporozoite protein (CSP) was determined in the crushed head and thorax by ELISA with monoclonal antibodies against *P. falciparum* CSP [29]. The infection rate was calculated as the proportion of positive mosquitoes to the total number of malaria vectors. The entomological inoculation rate was calculated as the infection rate multiplied by the human biting rate. A subsample of the *An. funestus* group was identified by the PCR analysis as described by Cohuet *et al*. [30].

### Statistical analyses

For each method, human biting rate (HBR) and CSP rate was calculated. Qualitative data were compared with the Pearson chi^2^ or Fisher exact test and quantitative data by non-parametric tests (Kruskal-Wallis). Statistical analyses were performed with Stata® 10.1. A *p* value of ≤ 0.05 was considered significant.

## Results

### Overall results

Between July 2011 and April 2013, during 264 person-nights, 288 *An. funestus* were collected by standard catching at night. During the same period, 143 *An. funestus* were captured by pyrethrum spray catches. Between January and April 2013, 78 *An. funestus* were captured by new catching by day during eight person-days. *Anopheles gambiae*, the commonest malaria vector, others *Anopheles* species vectors species such as *Anopheles pharoensis, Anopheles ziemanni, Anopheles coustani, Anopheles rufipes* and *Anopheles squamosus* and species of *Aedes, Culex* and *Mansonia* were also captured.

Of a subsample of 118 *An. funestus*, 66 were collected by standard catching at night, 22 by new catching by day and 30 by pyrethrum spray catches, PCR analysis confirmed that all were exclusively *An. funestus*.

### Human biting rate

**Figure 1:**
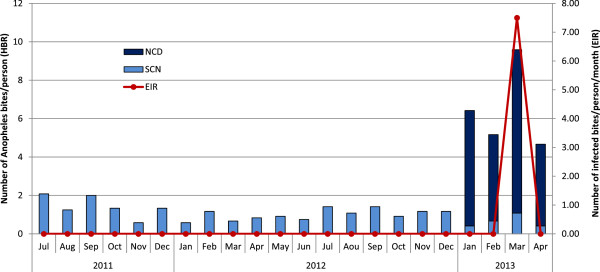
**Monthly human biting rate and entomological inoculation rate of *Anopheles funestus *calculated by standard catching at night (SCN) and new catching by day (NCD) after the second implementation of long lasting insecticide nets in Dielmo.** SCN represented the Standard Catching Night at 19:00 and 07:00 from July 2011 to April 2013; NCD represented the New Catching by Day at 07:00 and 11:00 from January to April 2013.

The monthly variations in the human biting rate (HBR) of *An. funestus* during the study period are shown in Figure 1. The rate remained low but stable, at fewer than two bites per person per night with SCN. When the collection was extended until 11:00 by NCD, the human biting rate was eight times higher than the average rate by SCN (Pearson chi^2^*p* < 0.001).

### Feeding time

**Figure 2:**
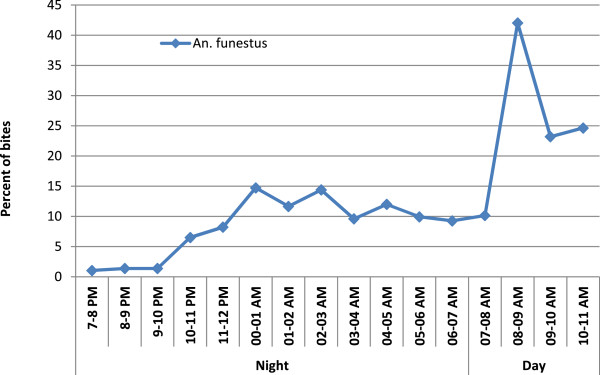
**Trends in the biting cycle of *Anopheles funestus *** **night and dayly human landing catche after the implementation of LLINs (cumulated number of bites of *An. funestus *** **per hour by total number of bites per night x 100).**

**Figure 3:**
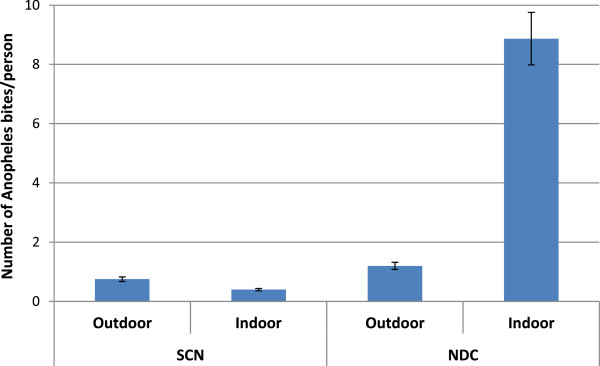
**Number of *An. funestus *bites per person (% and 95% confidence interval) according to a hour catch.** SCN represented the standard catching at night between 19:00 and 07:00 from July 2011 to April 2013; NCD represented the new catching by day between 07:00 to 11:00 from January to April 2013.

The hourly aggressiveness of *An. funestus,* as classically described, increased after night fall to a maximum around 02:00 and then decreased until the morning (Figure 2). Increasing aggressiveness was observed, however, between 07:00 and 11:00, corresponding to the time when people are not under LLINs but involved in early household and farming activities. A significant difference in *An. funestus* aggressiveness was observed between standard catching at night and new catching by day (Kruskal-Wallis test *p* < 0.0001). A significant difference in endophilic rate was observed in *An. funestus* captured in daylight (new catching by day) in comparison with standard catching at night (Pearson chi^2^*p* < 0.0001) (Figure 3).

### Parity rate and human blood index

The mean parity rate was 93.1% (n = 188; CI: 88.4-96.2) for *An. funestus* captured by standard catching at night and 85.7% (n = 70; CI: 75.2-92.9 by new catching by day. There was no significant difference between parity rates (Pearson chi^2^ = 3.4; *p* = 0.065). In an analysis of the blood meal origin for *An. funestus* sampled by PSC between July 2011 and April 2013 (n =84), the proportion that had fed on humans was 73.8% (CI: 63.0–82.8).

### Infection rate

The *P. falciparum* infection rate, measured monthly by the presence of sporozoites in the salivary glands (by ELISA), did not change during the study period and was null for mosquitoes captured by standard catching at night (n = 288), whereas one mosquito was found positive for the CSP antigen among the 78 specimens captured by new catching by day and, giving a mean CSP rate of 1.28%.

## Discussion

In Africa, *An. funestus* plays a major role in malaria transmission in several areas [31,32]. The *An. funestus* group is composed of many species, which can be differentiated only from discrete traits of both larvae and adults [33]; specific PCR has been developed to differentiate the species in the group [30]. Nine species have been described, with widely different biology and vectorial capacity. Apart from *An. funestus*, the other species appear to be mainly zoophilic [34]. Human *Plasmodium* has been found mainly in *An. funestus*, which is an excellent vector, with high vectorial capacity, and only rarely in *Anopheles rivulorum*[35]. *Anopheles vaneedeni* has been experimentally infested [36].

Most of the *An. funestus* in this study were captured between 07:00 and 11:00 in broad daylight. This new phenotype might have resulted from a shift in sibling species composition, but molecular analyses showed the same population of *An. funestus*, with a difference in biting activity after prolonged use of LLINs*. Anopheles funestus* populations captured after dawn are highly anthropophilic and endophilic, when most of the active population is occupied with household tasks, rustic employment and commerce. This is, therefore, a potential danger for vulnerable populations (children, pregnant women, patients and elderly individuals) who spend most of their time indoors.

*Anopheles funestus* is a major vector of malaria transmission in Dielmo [23,37]. The studies in the 1990s showed entomological inoculation rates that reached up 180 infected bites per person per year. In contrast, after introduction of LLINs, *An. funestus* almost completely disappeared, and the annual aggressiveness dropped from 17.2 bites per person per night to fewer than 1.2 (unpublished data). This may be due to the susceptibility of *An. funestus* to insecticides, an ineffective sampling method to estimate human exposure to mosquito bites or a behavioural change to avoid intra-domicilary control. In Africa, resistance of *An. funestus* to insecticides is widespread, although no *kdr* alleles has been found; nevertheless, the presence of biochemical resistance of this species to all classes of insecticide, pyrethroids, carbamates and DDT, has been demonstrated [38,39]. This phenomenon has not been seen in the village of Dielmo (unpublished data). However, inadequate capture methods may be the reason why this vector is not encountered. In a recent study in Zambia [40], it was observed that an Ifakara Tent Trap (model-C) placed outdoors was thirty four times more sensitive than HLC for sampling *An. funestus* after deployment of LLINs. In the case of *An. funestus* behavioural change, many studies have shown that new mosquito behaviour is an adaptive response to control strategies. Indeed the excito-repellents effects of treated bed nets repel mosquitoes outdoor, it follow a behavioural change strategy which is marked by changes in the biting time and place if the vector is highly endophilic and sometimes shift in host feeding [16]. In the current study, diurnal activity was seen in *An. funestus*, with a peak of aggressiveness between 08:00 and 9:00. These results are similar to those of studies in southern Benin after scaling up of universal coverage with LLINs, where 26.4% of *An. funestus* were caught after 06:00 [22]. The authors concluded that this vector has exophilic behaviour, because the villagers wake up early to work on crops. Exophilic behaviour was also reported in Tanzania by Russel *et al*. [13] after massive deployment of LLINs, although early biting activity of *An. funestus* was demonstrated. The authors showed that this behavioural change in *An. funestus* was responsible for residual outdoor transmission of malaria. Previous studies reported that behavioural changes to others *Anopheles* vectors. In Dielmo, Trape *et al.*[10] reported an early biting of *An. gambiae* after prolonged use of LLINs and in Benin Corbel *et al.*[11] showed greater exophagy rates of *An. gambiae* and *An. funestus* was recorded with a massive presence coupled with a shift from endophagic to exophagic behaviour. Another sibling species of *An. gambiae*, *Anopheles arabiensis* showed early biting activities in Ethiopia after use of LLINs both indoors and outdoors; and 80 per cent of this vector were captured before 22:00 with a peak activity between 19:00 to 20:00 [17]. All these studies showed the emergence of the behavioural adaptation of mosquitoes in response to indoor interventions, which can thus jeopardize the efficacy of these tools and also constitute a risk to people who are so accessible.

In the present study, one mosquito was found infected, which was captured during the day, while since distribution of impregnated nets in this village, no *An. funestus* had been found positive by ELISA. This constitutes a real risk for malaria transmission in this locality. The new phenotypes of mosquito have a human blood index and high parity, showing that a close relationship has been maintained between *Anopheles* and humans, despite the distribution of LLINs. The concept of phenotypic plasticity or the selection of specific genetic traits for adapting to environmental conditions appears to confirm Pates *et al*. [21]. This behavioural change represents a characteristic to respond to new environmental conditions represented by the use of LLINs. Russell *et al.*[41] showed in Tanzania that *An. funestus* was strongly exophilic and made a trophic deviation to cattle after universal coverage with LLINs, while in Benin [22] and Senegal, an exceptional, remarkable adaptation has been seen. The fundamental concept of biology appears to be respected: in the face of stressful situations, organisms adapt or disappear [42].

## Conclusion

Control strategies against malaria, such as indoor residual spraying and LLINs, represent powerful, appropriate tools, as African malaria vectors were considered to be entirely endophilic and anthropophilic. This study suggests *An. funestus* has adapted its biting time to the new situation (use of impregnated nets). Behavioural change in response to insecticides may make control tools ineffective, thus negatively affecting malaria control strategies. This remarkable behavioural adaptation of mosquitoes to insecticide-based vector control interventions requires heightened, close awareness in the context of pre-elimination of malaria.

## Competing interests

The authors declare that they have no competing interests.

## Authors’ contributions

MON performed field work. SMD performed laboratory work with assistance of SS. MON analysed the data. SS, MON and JFT drafted the manuscript. SD, ND, PMS and MH provided substantial improvement of the manuscript. CS and JFT provided scientific supervision of Dielmo project. MON planed the study design. All authors read and approved the final manuscript.
